# Editorial

**Published:** 1876-09

**Authors:** 


### Editorial.

### THE CONTROVERSY BETWEEN MR. JONATHAN HUTCHINSON AND DR. TILBURY FOX.

In the third fasciculus of the Illustrations of Clinical Surgery which are now in course of publication, and which represent the results of Mr. Hutchinson's professional labors for the last twenty years, there is a plate which has attracted unusual attention. It is a portrait of a disease which is there termed Cheiro-Pompholyx; the illustration being accompanied by text in which the history of the case is detailed. Soon after the appearance of this fasciculus, the 8th part of Dr. Fox's Atlas of Skin Diseases was placed in the hands of the profession, and not a few were surprised to find on its 48th page the following "Note on Cheiro-Pompholyx:"

"Mr. Hutchinson has recently described and figured an eruption which he has termed cheiro-pompholyx, without, as he remarks, 'intending to imply a relationship to true pemphigus,' by that name, which is therefore a most unfortunate and misleading one. This eruption is nothing more nor less than that of which I first gave a full account in 1873, in the third edition of my work on skin diseases, under the designation of Dysidrosis; and the case from which Mr. Hutchinson's representation was taken was an exaggerated example of dysidrosis that came under my observation, and was sent to me by Dr. Russell Reynolds. The disease is not a pompholyx. It is moreover not limited to the hand, and therefore not entitled to be styled by the prefix cheiro. This disease is not produced by effusion of serum into the skin, as Mr. Hutchinson declares, but by distension of the sweat apparatus, and uplifting of the cuticle by sweat and the production of large vesicles or bullæ by the free secretion of sweat and the coalescence of smaller vesicles. I shall give further details when I come to represent the disease and to describe it further on; but it is strange that my original description of the disease should have been ignored."

Mr. Hutchinson in the text accompanying his illustration, admits that his patient was seen by Dr. Fox in the year 1869. The former has more recently published (*London Lancet*, July, 1876,) an article which may be presumed to embody his rejoinder to Dr. Fox's note. It is entitled, "Cheiro-Pompholyx—Notes of a Clinical Lecture on 'A recurrent bullous eruption on the hands,' written out in April, 1871, and now printed verbatim."

This is not the first occasion upon which two eminent men have contended for the honor of a lady's hand. What are the real merits of the issue? Viewing the case comprehensively, it becomes clear that to Mr. Hutchinson is due the credit of having first seen and described the lesions, as well as of appreciating their interesting features to the extent of ordering the first drawing of the hands executed. His colored plate of the disease is admirably finished, and incomparably superior to those which Dr. Fox is now editing.

The verbal descriptions of the malady, as given by both authors, are equally good. This is indeed nothing more than we have a right to expect, having in mind the recognized ability of the two gentlemen, and the fact that each was engaged in portraying precisely the same objective phenomena. We have had an opportunity of studying less exaggerated forms of the disease than that delineated, and are thus in position to know that the comparisons instituted by the two, (in which, curiously enough, often the same words are employed,) are strikingly accurate.

*Per contra*, it is to be remarked that Dr. Fox has, without question, established the correct pathology of the lesion; and to him is due the honor not only of this, but of appropriately and accurately naming the disease. The designation given by Mr. Hutchinson has about it the flavor of a musty antiquity. It recalls the "ancient and fish-like odor" of the period when a constellation in the heavens, as well as a skin disease, had a crab for a god-father. "Pompholyx" is a relic of the literature of Hippocrates, Celsus and Avicenna. And yet, by this term Mr. Hutchinson does "not intend to imply a relationship to true pemphigus." It would be curious and interesting to learn the author's idea of the distinction between pompholyx and true pemphigus. Etymologically considered, one name likens a bulla to a blister, and the other to a bladder! The clinical distinction between the two, (if they are two,) is just about as definite. It is probably for this reason that the term pompholyx has nearly disappeared from the literature of cutaneous medicine. It is not to be found in the description of a separate disease in any of the more recent text-books, and is entirely omitted in Hebra's classification. The term pemphigus, on the other hand, is not only respectable for its antiquity, but has become prolific in its old age. Von Martius, in 1829, described ninety-seven varieties of the species, and Wilson mentions fifteen. It has been made to designate a wide range of disorders of the skin, from those produced by syphilis to certain curious tumors, having caseous contents, found upon the integument of a child. There has been too much of this unwarrantable use of names for each separate symptom displayed upon the skin. It has been the reproach of dermatology that it consisted of a nomenclature.

We may even go further than Dr. Fox, who objects to the prefix cheiro, on account of the non-limitation of the disease to the hand. For, while Mr. Hutchinson describes a red lichenous rash over the body of the patient, he does not attempt to explain its occurrence. But if Dr. Fox's pathology be correct, it can be readily understood how the rapid and free secretion of retained sweat, (forming bullæ upon the hands,) might be accompanied by hyperæmia of the sudoriparous follicles of the body elsewhere, producing an eruption quite similar to that of lichen tropicus.

Our object in calling attention to the publicly expressed difference of opinion of these two distinguished medical men is, in part, to commend the manner in which they have thus far discussed that difference. There have been no acrimonious charges, no offensive personal allusions in their written expressions. In other words, they have not only shown that they were gentlemen, but members of a profession whose obligations required their sustaining such relations to each other as the public would recognize and respect. Scientific discussions can have but one object — the ascertaining of the truth. The question between two savants will always be, not "Which of us is in the right?" but, "What is the right, even if we be both wrong?" Thus conducted, every discussion has a value. Conducted with any other end in view, it becomes a public impertinence.

Dermatologists have a worthy precedent in these matters. In the now historical contest between Professors Hebra, of Vienna, and Pick, of Prague, on the question of the parasitic or non-parasitic nature of eczema marginatum, (Archiv. für Dermatol. u. Syph. I. 1, 2, and 3,) there was great difference of opinion combined with a spirit of fairness and courtesy, and an inflexible purpose to know the truth. The result was, that to-day the real character of the disease they had under consideration is distinctly recognized.

---

### ARCHIVES OF CLINICAL SURGERY.

Such is the name of a new Monthly Journal to be devoted to the different departments of surgery exclusively. The initial number is before us. It has a very full table of contents and is neat and creditable in appearance. We are glad to see it so early give promise of a fulfillment of its pledge to give regular reports from the surgical wards of the hospitals in all our larger cities. But some of the hospital reports need to be made fuller and more complete to be valuable. While it is true that in reports of cases we only need a statement of essential facts to make them valuable for subjects of study and comparison, it is nevertheless true that nothing less than all the cardinal points will suffice.

Dr. Edward J. Birmingham is the editor, and Rutledge & Co. the publishers (102 W. 49th St., New York), and they insist on having four dollars in advance for each subscription. We shall exchange compliments with the new Journal every month and with great pleasure.

